# Discovery of a Potent Candidate for RET-Specific Non-Small-Cell Lung Cancer—A Combined In Silico and In Vitro Strategy

**DOI:** 10.3390/pharmaceutics13111775

**Published:** 2021-10-24

**Authors:** Priyanka Ramesh, Woong-Hee Shin, Shanthi Veerappapillai

**Affiliations:** 1 Department of Biotechnology, School of Bio Sciences and Technology, Vellore Institute of Technology, Vellore 632014, India; priyanka.r@vit.ac.in; 2 Department of Chemical Science Education, College of Education, Sunchon National University, Suncheon 57922, Korea; 3 Department of Advanced Components and Materials Engineering, Sunchon National University, Suncheon 57922, Korea

**Keywords:** LC-2/ad cell line, drug discovery, docking, MM-GBSA calculation, molecular dynamics, cytotoxicity assay

## Abstract

Rearranged during transfection (RET) is a tyrosine kinase oncogenic receptor, activated in several cancers including non-small-cell lung cancer (NSCLC). Multiple kinase inhibitors vandetanib and cabozantinib are commonly used in the treatment of RET-positive NSCLC. However, specificity, toxicity, and reduced efficacy limit the usage of multiple kinase inhibitors in targeting RET protein. Thus, in the present investigation, we aimed to figure out novel and potent candidates for the inhibition of RET protein using combined in silico and in vitro strategies. In the present study, screening of 11,808 compounds from the DrugBank repository was accomplished by different hypotheses such as pharmacophore, e-pharmacophore, and receptor cavity-based models in the initial stage. The results from the different hypotheses were then integrated to eliminate the false positive prediction. The inhibitory activities of the screened compounds were tested by the glide docking algorithm. Moreover, RF score, Tanimoto coefficient, prime-MM/GBSA, and density functional theory calculations were utilized to re-score the binding free energy of the docked complexes with high precision. This procedure resulted in three lead molecules, namely DB07194, DB03496, and DB11982, against the RET protein. The screened lead molecules together with reference compounds were then subjected to a long molecular dynamics simulation with a 200 ns time duration to validate the inhibitory activity. Further analysis of compounds using MM-PBSA and mutation studies resulted in the identification of potent compound DB07194. In essence, a cell viability assay with RET-specific lung cancer cell line LC-2/ad was also carried out to confirm the in vitro biological activity of the resultant compound, DB07194. Indeed, the results from our study conclude that DB07194 can be effectively translated for this new therapeutic purpose, in contrast to the properties for which it was originally designed and synthesized.

## 1. Introduction

Targeted therapies using tailored inhibitors against oncogenic driver kinases have transformed the landscape of cancer management, including non-small-cell lung cancer (NSCLC) [1]. Notably, first-generation inhibitors against oncogenic drivers such as gefitinib, erlotinib (EGFR mutations), and crizotinib (ALK rearrangement) have established a novel treatment paradigm for the use of targeted inhibitors in genetically defined NSCLC patients [2,3]. Despite the earlier success of these strategies, the emergence of acquired resistance against the therapy has become a significant challenge in developing selective and more potent next-generation inhibitors.

Rearranged during transfection (RET), a transmembrane tyrosine kinase receptor was found to be overexpressed in 1–2% of never-smoking NSCLC patients [4]. In general, it plays a vital role in the development of neural crest cells in the nervous system and kidney morphogenesis. RET consists of three domains: adhesion, tyrosine kinase, and extracellular domain. Activation of RET involves autophosphorylation of a fusion protein complex with a glial cell line derived from neurotrophic factors (GDNF) and GFR-α, a cell membrane-bound coreceptor [5,6]. The downstream signaling of RET assists in cell migration, proliferation, and differentiation. Nevertheless, genetic alteration of RET oncogenes promotes ligand-independent activation of driver kinases, resulting in tumorigenesis. A study in late 2011 revealed that pericentric inversion, rearrangement, dimerization, and activation of RET proteins with KIF5B and CCD6C in NSCLC were analogous to the mechanism of ALK [7]. Multiple Kinase Inhibitors (MKIs), including cabozantinib and vandetanib, gave the first glimmer of hope for the treatment of RET-positive NSCLC patients. However, these nonselective MKIs demonstrated limited response durability and off-target side effects in NSCLC patients [8]. Thus, selective inhibitors such as selpercatinib and pralsetinib were developed to offset the debility of the multiple kinase inhibitors.

Recently, the emergence of solvent front mutations and gatekeeper mutations in RET-positive NSCLC patients has been reported as the primary cause for the development of acquired resistance against the targeted kinase inhibitors [9]. A similar pattern of the solvent front and gatekeeper mutations was observed in several types of oncogenic driven NSCLCs. A typical example of other proteins associated with resistance in NSCLC includes ALK rearrangement, ROS-1 positive, and EGFR mutations. A significant number of reports are available to tackle resistance caused by the above genes [10]. However, studies on RET mutations in NSCLC are very minimal and are not satisfactory [11]. In addition, it is to be noted that MKIs were the only choice of drug to treat RET-driven NSCLC. Recently, the selective inhibitor pralsetinib was administered in both naïve and platinum-based chemotherapy-treated patients. Among the cohort, 10% of the patients were detected with solvent front mutations (G810C/S), 15% were detected with MET amplification and 5% of the cohort were detected with KRAS amplification [12]. Although the study ended up with satisfactory results and was found to have overcome gatekeeper mutations during the clinical trials, the adverse side effects of the drug limit its efficacy and it failed to overcome solvent front mutations [13]. Moreover, the resistance mechanism of solvent front mutations to selective inhibitors is not yet reported in the literature [14]. Hence, developing next-generation targeted kinase inhibitors particularly against RET solvent front mutations is desperately needed to overcome the acquired resistance.

Virtual screening of active compounds for hit identification and lead optimization has been made possible by advancements in bioinformatics and computer modeling in modern drug research [15]. For instance, Misra et al. identified two potent human great wall kinase inhibitors using the ZINC database that mitigate mitotic division in various types of cancer [16]. Similarly, Tamta et al. identified and validated three natural inhibitors against Mpro of SARS-CoV-2 using different in silico strategies including molecular docking, dynamics and MM-PBSA analysis [17]. In view of the successful evidence mentioned above, we implemented an integrated approach using pralsetinib as the reference inhibitor towards the screening of potent candidates against RET protein. Three different models were generated for performing a virtual screening process using FDA approved, experimental and investigative subsets of the DrugBank database, followed by docking analysis, to identify potent and highly selective RET inhibitors. The combined assessment in this study provides a highly potent drug-like candidate tailored for RET oncogenic drivers that can overcome acquired resistance in NSCLC patients.

## 2. Materials and Methods

### 2.1. Dataset Retrieval and Structural Refinement

The 3D conformation of RET tyrosine kinase with PDB ID: 2IVU and resolution of 2.5 Å were retrieved from Protein Data Bank (PDB) (www.rcsb.org/pdb, accessed on 27 August 2021). RET protein was prepared using the protein preparation wizard of the Schrödinger suite [18]. This process involves eliminating water molecules and impurities and incorporating hydrogen bonds and ionization states to the protein. The optimization and minimization of 2IVU were performed using the optimized potential for liquid simulation _2005 (OPLS_2005) force field, to increase the protein’s binding efficiency during docking analysis.

Table S1 (see Supplementary Materials) represents the existing RET inhibitors retrieved from various literature. They were utilized for pharmacophore hypothesis generation [19,20,21]. In addition, the spatial data file (SDF) of molecules in a different subset of the DrugBank repository containing a total of 11,808 compounds was extracted for proceeding with standalone library generation and the virtual screening process. The existing inhibitors and generated library were refined by attaching the hydrogen bonds, generating the stereoisomer, and identifying the significant ionization state using the LigPrep module of Schrödinger. Finally, the OPLS_2005 force field was used to optimize the ligand structures considered in our study [22].

### 2.2. Hypothesis Generation and Molecular Docking

The screening hypotheses were generated based on three different approaches, such as ligands, protein structure, and energetics of protein–ligand interactions with the aid of the Phase module of Schrödinger (version 5.3). Initially, the reference ligands were divided into actives and inactives based on their IC_50_ values (Table S1, see Supplementary Materials). Compounds with IC50 values higher than 5.0 μM were classified as inactive molecules. Consequently, the ligand-based pharmacophoric hypothesis was generated based on the common features of the active ligands using a tree-based partitioning algorithm [23]. Each common pharmacophore hypothesis (CPH) undergoes a rigorous scoring function based on alignment score, volume score, and vector score of the active ligands. The best CPH with high survival score was chosen for the virtual screening analysis. In the e-pharmacophore strategy, CPH was generated by docking the reference ligand pralsetinib and by mapping the energetic scores onto the atoms [24]. Similarly, receptor cavity-based CPH was developed based on the potential binding site of the RET protein using the SiteMap module of Schrödinger. Altogether, the chosen CPH contained four basic pharmacophoric features, namely a hydrophobic group (H), aromatic ring (R), hydrogen bond acceptor (A), and donor (D) [25]. Finally, the above-generated high precision CPH was used independently to screen the subsets of the DrugBank database. The resultant set in each screening was subjected to three hierarchical docking strategies, namely high-throughput virtual screening (HTVS), standard precision (SP), and extra precision (XP), which were implemented using the Glide module to identify the binders from nonbinders [26]. It is worth nothing that pralsetinib was used as the reference inhibitor throughout the investigation. Finally, the interaction pattern and the essential pharmacokinetic parameters such as stars, central nervous system response (CNS), and human oral absorption (HOA) were analyzed using the Qikprop module of Schrödinger.

### 2.3. Machine Learning-Based Standalone Rescoring Function

Random Forest score (RF score) based the rescoring function was implemented to determine the binding affinity between the ligand and RET for virtual screening using the open drug discovery toolkit available in https://github.com/oddt/rfscorevs, accessed on 27 August 2021. This scoring function is built using an RF algorithm with descriptors generated based on the distance between the atoms of the protein and the ligand that lie within 12 Å [27]. Compounds that have an RF score greater than the pralsetinib score were considered for further evaluation.

### 2.4. Chemical Similarity Calculations

Tanimoto coefficient (T_c_) was similarly calculated based on the MACCS fingerprint to evaluate the structural similarities of all the compounds. A higher value of T_c_ depicts the high structural similarity of the compounds with the reference molecule. Hence, the cut-off T_c_ value of >0.4 was considered in this analysis to quantify the fraction of compounds that exhibit structural similarity to pralsetinib [28]. In the present study, RDKit of the python library was implemented to generate the MACCS fingerprint and to calculate the T_c_ of the compounds.

### 2.5. Binding Free Energy and DFT Calculations

The prime module of the Schrödinger suite was used to determine the binding free energy of RET protein–ligand complexes. It is interesting to note that the binding free energies that were calculated using the MM-GBSA method correlated with the experimental study most of the time. The pose viewer file of the protein–ligand complex generated during Glide XP docking was used as a query for binding free-energy calculations. Further, the prime module utilizes the VSGB 2.0 solvation model to optimize hydrogen bonds, hydrophobic interactions, π–π interactions, and self-contact interactions [29]. The energy terms such as electronic interactions, Van der Waal’s interaction, entropy terms, polar and nonpolar contributions were considered for the binding free-energy calculations in the Prime package of Schrödinger.

Density functional theory (DFT) was calculated for the hit compounds obtained during the virtual screening process. Jaguar v8.7 was employed to calculate the nature of the interaction between the protein and ligand and molecular electrostatic properties such as highest occupied molecular orbital (HOMO) and lowest unoccupied molecular orbital (LUMO). Frontier orbital gaps of the hit compounds were calculated to analyze the kinetic stability and chemical activity [30].

### 2.6. Assessing the Stability and Binding Mode of 2IVU–Ligand Complex

A molecular dynamics (MD) simulation of the RET–ligand complex was used in this study to assess the stability and conformational changes of a protein–ligand complex. GROMACS v5.1.2 (Virginia Tech Department of Biochemistry, Blacksburg, VA, United States) with GROMOS96 43a1 force field was used for the simulation. The topology files and the parameters for the ligands were developed using the PRODRG server. Dodecahedron box with dimensions of 1 nm × 1 nm × 1 nm was configured using editconf inbuilt tool of GROMACS. Subsequently, the Simple Point Charge model was explicitly used for solvating the complex system in a dodecahedron box. During the solvation process, the system exhibited a total charge of +8. Hence, eight chlorine counter ions were added to neutralize the protein system. The weak Van der Waals linkages were removed using the Steepest Descent algorithm to minimize the energy of the complex. Electrostatic interactions were enlightened by applying the Particle-Mesh Ewald method. LINCS algorithm was implemented for constraining the hydrogen bonds and for truncating the Van der Waals interactions. The canonical calculations of NVT (Number of particles, Volume, and Temperature) and NPT (Number of particles, Pressure, and Temperature) ensembles were executed for restraining the position. The complex system was heated using a Berendsen thermostat at 300 K with a lapsing time of 0.1 ps and pressure of 1 bar. Precedent to MD simulation, a pre-run was performed with a 1000 kJ mol^−1^ nm^−2^ force constant as a positional restraint for 50 ps. Ultimately, final MD for the apoprotein (without ligand) and protein–ligand complex were carried out for 200 ns [31]. Trajectories for the complex system were saved every 2 fs. Root Mean Square Deviation (RMSD), Root Mean Square Fluctuation (RMSF), H-bond linkages, free-energy landscape, and the salt bridge between the ligand and the protein were also evaluated using GROMACS utilities. In essence, the MM-PBSA strategy was also implemented to calculate the empirical free energies between the RET receptor and the identified potential ligands with high precision [32].

### 2.7. In Vitro Analysis

The anticancer activities of the potential compounds together with pralsetinib were determined using MTT assay [33]. The LC-2/ad cell was purchased from the European Collection of Authenticated Cell Cultures (Catalogue number: ECACC 94072247, Merck KGaA, Darmstadt, Germany) and grown in high-glucose Dulbecco’s Modified Eagle Medium (AL149, Himedia, Mumbai, India) for 24 h. The cell line contains CCDC6-RET driver gene fusion isolated from the lung of a 51-year-old adenocarcinoma Japanese patient. This cell line is widely used to study intracellular signaling pathways, resistance mechanisms, and drug sensitivity against RET fusion in NSCLC samples. The chemical compounds pralsetinib and DB07194 were purchased from MolPort (Catalogue number: HY-112301, Molprot, Riga, Latvia) and Merck (Catalogue number: 574715-2mg, MercK KGaA, Darmstadt, Germany), respectively. Consequently, the grown LC-2/ad cells were exposed to reference and hit compound concentrations ranging from 6.25 µM/mL to 100 µM/mL for four days at 37 °C in a 5% CO_2_ atmosphere. The absorbance of the samples was read at 570 nm and 630 nm as the reference wavelength to correct the nonspecific background values. The experiment was performed in triplets, and the mean value of the assays was considered in our analysis. Finally, the IC_50_ of the compound was determined using a linear regression equation and viability graph. In addition, a statistical comparison of cell viability between control and drug candidates was carried out using one-way ANOVA. For all comparisons, a *p*-value of less than 0.05 was regarded as statistically significant.

## 3. Results and Discussion

### 3.1. Pharmacophore Modeling and Virtual Screening

A pharmacophore is a collection of chemical features and spatial properties required for the ligand to interact with a macromolecular target and elicit a biological response [34]. In the present investigation, about 193 ligand-based pharmacophore hypotheses were developed with the assistance of actives and inactives (Table S1, see Supplementary Materials) using the Phase module of Schrödinger (v5.3). Depending on the survival score, a five feature CPH containing one hydrogen bond acceptor (A), one hydrogen bond donor (D), one hydrophobic group (H), and two aromatic rings (R) were selected. Likewise, two other hypotheses, DHRRR and ADDHR, were generated from the e-pharmacophore and receptor cavity-based strategies, respectively. A total of 3673, 1198, and 4595 compounds were obtained after phase screening using pharmacophore, e-pharmacophore, and receptor cavity-based hypotheses, respectively. The screened compounds were subjected to three tiers of docking such as HTVS, SP and XP using pralsetinib (−7.79 kcal/mol) as a reference compound. In each stage, 50% of high-scoring leads were passed on to further analysis. This process yielded a total of 887 (Pharmacophore–208; e-Pharmacophore–103; Receptor cavity-576) compounds possessing better binding capability than the reference compound which were carried for further analysis.

### 3.2. Rescoring Methodologies

Random Forest scoring is a novel machine-learning algorithm implemented extensively in virtual screening to forecast binding affinity on a varied range of targets, using descriptors based on RF Score version v1-3. Despite being less precise on physicochemical properties, the RF scoring function typically outperformed conventional scoring systems in estimating binding affinity [35]. Hence, in the current investigation, rescoring was conducted using a random forest approach for all the hit molecules obtained in the screening process. The results from our algorithm depict that 500 out of 887 compounds had a higher RF score than pralsetinib. Further, T_c_ was calculated between the reference ligand and the hit molecules to measure the structural similarity [36]. The results indicate that 406 molecules were highly similar to pralsetinib with a T_c_ threshold value greater than 0.4. RF score and T_c_ values of compounds obtained from pharmacophore, e-pharmacophore and receptor cavity-based strategy are tabulated in Tables S2–S4, respectively (see Supplementary Materials). On comparing the RF score and T_c_ results of all the hit molecules, 78, 39, and 59 compounds were found to possess better similarities and RF scores, respectively from pharmacophore, e-pharmacophore, and receptor cavity-based strategies. The results from all three hypotheses were then integrated to eliminate false positive prediction. Notably, only 18 lead molecules were found to be in common among all the three approaches with high similarities and RF scores. The combined result of 18 lead molecules and their scores are tabulated in Table 1.

### 3.3. Postdocking MM-GBSA Analysis

The binding free energies of complexes were determined to validate the binding ability of the ligands to the target protein. The summary of the binding free energy of each complex is tabulated in Table 2. It can be observed that the ΔG_bind_ of the complexes varied from between −69.235 kcal/mol and −39.610 kcal/mol. Note that only eight compounds resulted in a binding free-energy value above −55 kcal/mol. The Van der Waals energy for all the compounds was observed to be highly favorable to the overall binding energy. The coulomb energy provided the second-highest contribution to the interaction in all the compounds; however, the high solvation energy compensated coulomb contribution in ΔG_bind_. The contribution of covalent energy is almost unfavorable or negligible to the binding of the compounds DB08583, DB07606, and DB04751. Additionally, ligand strain energy depicts the deformation of ligands during the interaction, which is considered one of the most important parameters during the MM/GBSA analysis [37,38]. It is clear from the table that almost all the predicted compounds undergo less deformation than pralsetinib during interaction with the target protein except DB08583, DB07606, and DB04751. Although, the compounds DB08583, DB07606, and DB04751 exhibited better binding free energy. Higher ligand strain energy decreases the binding efficacy of the compounds with target receptors. Eventually, DB07194, DB03496, DB11982, DB12672, and DB12848 showed more satisfactory Coulombic potential and ligand strain energy than the other compounds, facilitating tight binding to the RET protein. Of note, these compounds exhibited minimal covalent energy contributions towards ΔG_bind,_ a key factor for forming a thermostable complex with RET protein.

### 3.4. HOMO–LUMO Theory Analysis

**Figure 1:**
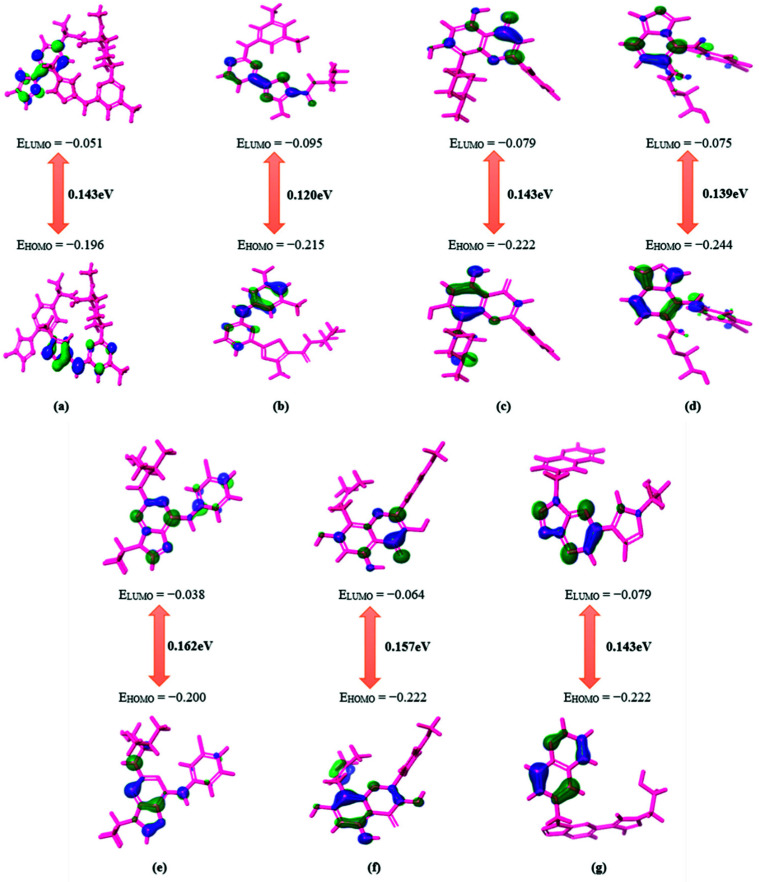
Graphical representation of HOMO–LUMO energy gap calculation for reference (**a**) Pralsetinib and hit compounds (**b**) DB07194, (**c**) DB03496, (**d**) DB11982, (**e**) DB04751, (**f**) DB12672 and (**g**) DB12848.

All five compounds with high binding free energy and lower ligand strain energy were optimized using B3LYP-D3 theory and LACVP++ basis set (Schrödinger, Bangalore, India). Since the reactivity of a compound is directly related to the energy gap, the parameters HOMO and LUMO had are significant [39]. The molecule with a minimal energy gap between frontier orbitals is usually accompanied by a substantial chemical reactivity and weak kinetic stability which depicts the highly favorable potential reactions [40]. The energy gap between HOMO and LUMO is shown in Figure 1. It is observed that DB07194, DB03496, and DB11982 exhibited a lower or equivalent gap to pralsetinib than DB12672 and DB12848. These results imply that compounds such as DB07194, DB03496, and DB11982 exhibit better biological activities than pralsetinib.

### 3.5. Interaction Pattern and Pharmacokinetic Analysis

The interaction pattern of hit compounds in the binding pocket of the receptor is represented in Figure S1 (see Supplementary Materials). On analyzing the binding pattern of pralsetinib, two hydrogen bonds were found between the cyclohexane carbomide group and the ALA807 residue of RET, and one additional hydrogen link was observed between the pyridine ring of pralsetinib and the SER811 residue of the protein. The ligand interaction diagram of DB07194 clearly shows the formation of two hydrogen bonds between the amino pyrimidine group of DB07194 and the residues ASN879, ASP892 of the RET protein. Likewise, the N-methylpiperidinyl and flavone group of DB03496 displayed hydrogen bonds with ARG878 and ALA807 residues of the receptor. In addition, a salt bridge was formed between the tertiary amino group of N-methylpiperidinyl and the residue ASP892 in the DB03496-RET complex. In the case of DB11982, a hydrogen bond formation between the pyridine carboxamide and ARG878 of RET protein was observed. It is interesting to note that the anticancer property of these functional groups of the hit compounds involved in the interaction with the RET protein has been reported recently [41,42,43]. The existence of interactions by the key residues ASN879 and ASP892 of hydrophobic pockets in RET proteins has also been observed in the other approved drugs, crizotinib, and sorafenib, respectively. The interaction pattern of the drugs is given in supplementary Figure S2.

Furthermore, the essential pharmacokinetic parameters were analyzed to prevent the elimination of the compounds during clinical trials in the future. Table S5 (see Supplementary Materials) characterizes the interaction patterns and pharmacokinetic features of the lead compounds. The hit compounds displayed satisfactory pharmacokinetic and pharmacodynamics properties. Of note, key properties such as solubility, blood–brain barrier, stars, human oral absorption, and CNS activity were found to be in the acceptable range Stars denote the number of pharmacokinetic features that lie outside the required range. Interestingly, none of the hit compounds were found to have outliers based on the star values. Moreover, the capability of stimulating the central nervous system response by the hit molecules was comparatively similar to pralsetinib (CNS = −2). Undeniably, the HOA of all the predicted molecules was higher than pralsetinib (HOA = 2), which shows the efficacy of a drug that can be attained easily through oral administration in humans.

### 3.6. Protein–Ligand Complex Stability Analysis

**Figure 2:**
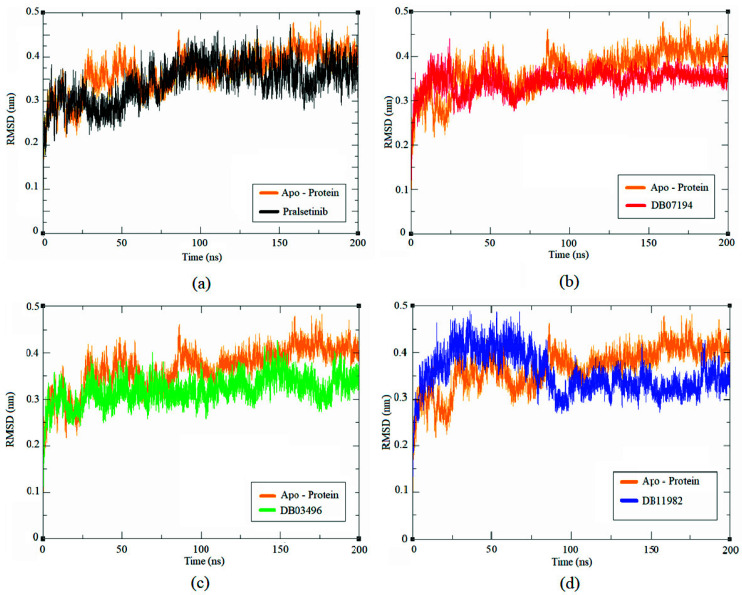
Time evolution of RMSD values for the apoprotein and protein–ligand complexes: (**a**) Pralsetinib, (**b**) DB07194, (**c**) DB03496 and (**d**) DB11982.

The stability and dynamic characteristics of protein-lead inhibitor complexes were investigated using MD simulations. It provides precise insights on protein–ligand interactions, allowing for the visualization of the influence of ligand binding on protein and its contribution to their stable, bound conformation [32]. The RET protein complexed with three hit compounds alongside the reference complex was analyzed using 200 ns MD simulations. The extent of deviation of atoms in the protein-lead complex during the simulation process is explained using RMSD plots. It is interesting to note that the obtained results correlate well with our initial findings. The results are shown in Figure 2a–d. Figure 2 reveals that all the compounds showed an increased RMSD deviation within the interval of 0–30 ns simulation time. A minimal deviation in the pattern was observed between 30 ns and 75 ns. Consequently, all the compounds maintained a stable equilibrium of ~0.30 nm from 75 ns to the end of the simulation process. Towards the end of the simulation, minimal RMSD values of 0.345 nm, 0.323 nm, and 0.371 nm were observed for DB07194, DB03496, and DB11982, respectively, smaller than pralsetinib (0.385 nm) and apoprotein (0.414 nm). In all the cases, the RMSD data corresponding to apoprotein was significantly higher than the ligand-bound structure investigated in our analysis. This suggests that the hit compound could adapt to a more stable conformation than pralsetinib in the binding pocket of RET protein. Moreover, the overall deviation of hit molecules was less than ~5 nm, depicting the stability of the RET protein in the presence of lead molecules. Thus, we hypothesize that the predicted DB07194 compound could have a higher inhibitory potential against RET protein than pralsetinib.

Guterres and Im showed that active compounds have less RMSD than inactive compounds in 100 ns MD simulations [44] From the DUD-E set, they randomly selected 56 targets. For each target, 10 compounds, five actives and five decoys were selected. They observed that the active compounds have a unimodal RMSD distribution centered at 4 Å, whereas the decoys have a skewed-right distribution, showing that a lot of them leave the binding pocket during the simulation. As mentioned, our molecules including pralsetinib have RMSD ~0.3 nm, which is consistent with the work of Guterres and Im. This implies that the three compounds could act as active compounds.

### 3.7. Residue Mobility Analysis (RMSF)

**Figure 3:**
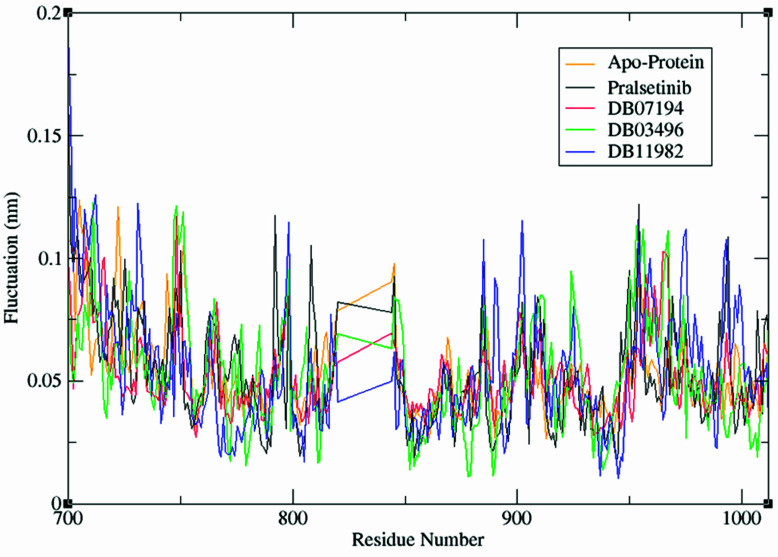
RMSF values for apoprotein and the protein–ligand complexes system during MD simulations.

RMSF depicts the flexibility of protein residues within the protein–ligand complex. As demonstrated in Figure 3, a similar pattern of fluctuation in the backbone was observed among all four systems. The region between Val871–Asp898 exhibited the least fluctuation, with less than ~0.05 nm, indicating the contribution of these residues to stable binding of predicted inhibitors with the RET receptor. Notably, important residues such as Asn879 and Asp892 showed fluctuations of ~0.04 nm, which were found within the conserved interaction region. It is to be noted that the presence of a highly stable protein–ligand complex was due to the formation of hydrogen bonds between these residues and the inhibitors. The other residues, Met700–Lys722 and Pro957–Arg982, showed high flexibility of about ~0.1 nm, suggesting that these residues contributed less to the RET–ligand interaction. These results are correlated well with the ligand interaction pattern discussed earlier. Moreover, a lower RMSF value depicts the well-organized region whereas a high RMSF value indicates loosely structured terminal ends of the complex [33]. In the present study, the apoprotein exhibited an RMSF value of 0.0696 nm whereas the complexes RET–Pralsetinib, RET–DB07194, RET–DB03496, and RET–DB11982 showed 0.069, 0.0665, 0.0773, and 0.033 nm RMSF values, respectively. The RET–DB07194 and RET–DB11982 complexes showed decreased RMSF values in comparison with the apoprotein and RET–pralsetinib complexes. This clearly depicts that the binding of lead molecules resulted in decreased flexibility of the catalytic residues. Hence, the identified lead compounds were very well positioned in the binding pocket of RET protein compared with other compounds considered in our analysis.

### 3.8. Hydrogen Bond Analysis

**Figure 4:**
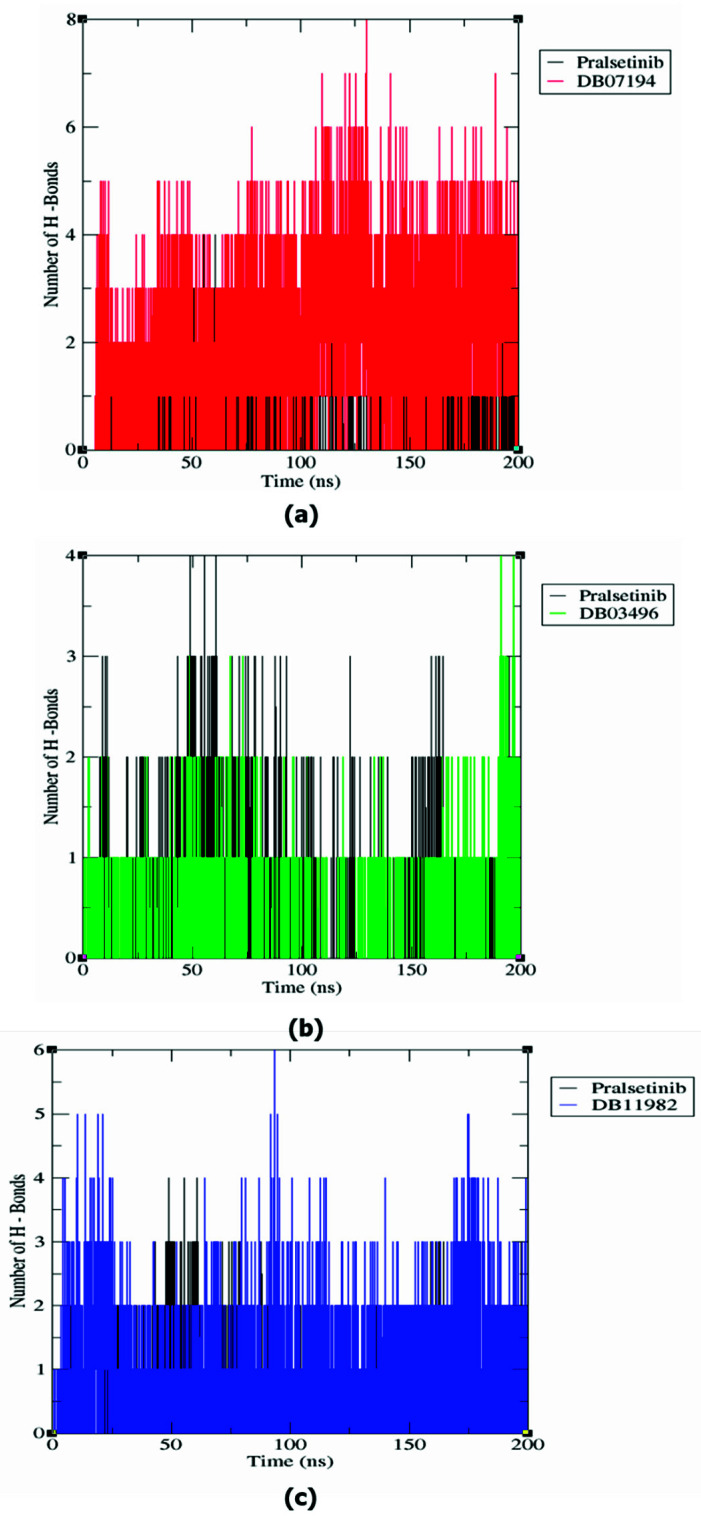
Comparative H-Bond analysis of pairs within 0.35 nm of the complex structures from MD simulation: (**a**) Pralsetinib and DB07194, (**b**) Pralsetinib and DB03496, and (**c**) Pralsetinib and DB11982.

The stability of a protein–ligand complex is usually analyzed based on different types of transient interactions, including electrostatic interaction, Van der Waals, hydrogen bonds, and many others [45]. Among them, the hydrogen bond is regarded as an important transient interaction facilitating the binding of ligands with protein. The existence of hydrogen bonds in the complex structures was calculated from the MD trajectory. The RET–DB7194, RET–DB03496, and RET–DB11982 showed 0–8, 0–4, and 0–6 H-bonds, respectively (Figure 4). These observations demonstrated that the predicted hits showed a higher number of H-bonds than the reference drug during simulation. From the results of the H-bond analysis, it can be concluded that the predicted compounds form a more stable interaction with the RET protein than pralsetinib.

### 3.9. Free Energy Landscape (FEL)

**Figure 5:**
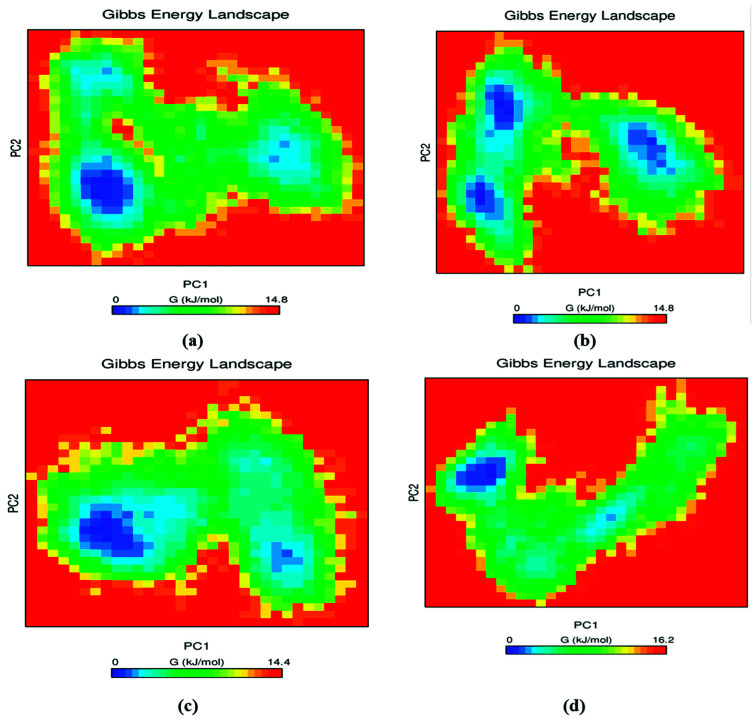
Contour plot demonstrating free energy landscapes of (**a**) RET–Pralsetinib, (**b**) RET–DB07194, (**c**) RET–DB03496 and (**d**) RET–DB11982 during 200 ns MD simulation.

An inbuilt GROMACS tool gmx_sham was employed further to investigate the conformational stability of the protein–ligand complex. The exchange of heat in a closed protein–ligand complex system is measured in Gibbs free energy [46]. This analysis provides information on energy minima confirmation and molecular fluctuation. Initially, the covariance matrix containing the eigenvalues was constructed using gmx_covar tool of GROMACS. Subsequently, the eigenvectors were obtained by diagonalizing the constructed matrix. Finally, the first two principal components (PC 1 and PC2) mapping the eigenvector to its corresponding eigenvalues were obtained using gmx_anaeig tool [47]. Figure 5 was plotted using the obtained PC1 and PC2, demonstrating the free energy landscape of the complexes. A dark blue color corresponds to the energetically stable and energy-minima favored complex conformation whereas a yellow color demonstrates the unfavorable conformation. The deep energy basin observed during the MD simulation process indicates the high stability of the complex system, while the shallow basin denotes the lower stability of the complex. The RET–pralsetinib complex contained two connected energy minima and one distinct energy minima. In the case of RET–DB03496 and DB11982, one deep energy basin as well as one shallow energy basin was observed, whereas, in the case of RET–DB07194, three deep energy basins were observed. Moreover, the Gibbs free energy of the two compounds (DB07194 and DB03496) was 14.8 kJ/mol and 14.4 kJ/mol, respectively, which were similar to the Gibbs free energy of pralsetinib (14.8 kJ/mol). Nevertheless, the Gibbs free energy of DB11982 was higher (16.2 kJ/mol) than the other two complexes. From Figure 5, it is evident that the energy basins were broad, clear, and distinct in all three compounds, and exhibited lower Gibbs free energy, which shows the stable confirmation of all three protein–ligand complexes.

### 3.10. MM-PBSA

The binding free energy analysis of the three hit compounds and the reference molecule were calculated using the trajectories pulled out from the last 10 ns of the simulation process. The binding energy for RET–pralsetinib (−9.445 ± 65.091 kJ/mol), RET–DB07194 (−111.920 ± 17.179 kJ/mol), RET–DB03496 (−74.514 ± 77.458 kJ/mol) and RET0–DB11982 (−37.949 ± 42.465 kJ/mol) were demonstrated in Table 3. RET–DB07194 exhibited a stable conformation with the least binding energy among all other compounds screened from our study. The total binding energy is composed of Van der Waals energy, electrostatic energy, polar solvation, and solvent accessible surface area energy. Among them, Van der Waals energy has the highest contribution to the overall binding energy, followed by polar solvation energy, SASA, and electrostatic energy, respectively. It is to be noted that the estimated pattern of binding free energies was similar to that of the MM-GBSA strategy. The predicted binding energies were well correlated with RMSD and hydrogen-bond analysis.

### 3.11. In Silico Evaluation of Lead Compounds against Point Mutant RET Receptor

As reported by Solomon et al., point mutations at different locations of RET resulted in the development of acquired resistance against the existing inhibitors. Specifically, the development of resistance due to solvent front mutations prevented the inhibitors from accessing the binding pocket of the protein [9,10]. Hence, we evaluated the binding capability of lead compounds against the mutant RET receptor using docking studies and MM-GBSA analysis. The results of docking and MM-GBSA analysis are tabulated in Table S6 (see Supplementary Materials). About 11 points mutated the RET-protein structure, containing 4 point mutations at the gatekeeper region, 4 mutations at the solvent front region, and 3 mutations at other regions, were generated using the homology modeling suite of Schrödinger. The docking analysis of the three lead compounds against RET mutants revealed that DB07194 had overcome G810C and G810V solvent front mutations with higher binding free energy than pralsetinib and the other two hit molecules. On the other hand, the compound DB03496 exhibited significant inhibitory activity against the G810R solvent front mutation. In addition, both the compounds DB07194 and DB03496 inhibited M918T mutation with high binding free energy, at −74.11 kcal/mol and −87.16 kcal/mol, respectively.

In some cases, including V804M mutational study, all the three lead compounds exhibited a high docking score. In contrast, the binding free energy of the lead compounds was lower than the pralsetinib, preventing them from overcoming resistance. Unfortunately, DB11982 did not overcome the acquired resistance in any RET mutant structures investigated in our study. On analyzing the interaction pattern of DB07194, three hydrogen bonds formed between the amino pyrimidine group and ARG874, ARG878, ASN879 had assisted the compounds in overcoming the acquired resistance caused by the G810C mutation in RET. Interestingly, a similar pattern of interaction was observed against the G810V mutation. In the case of M918T mutation, three hydrogen bonds were formed between DB07194, SER811 and ALA807 of the RET protein. Overall, DB07194 showed higher inhibitory activity against RET mutants, including G810C, G810V, and M918T, than DB03496 and DB11982. It is to be noted that pralsetinib has a comparatively lower potential than DB07194 to overcome the solvent front mutation, which might be due to the absence of an amino pyrimidine group in its structure.

### 3.12. Cell Viability Analysis of DB07194 against LC-2/ad

**Figure 6:**
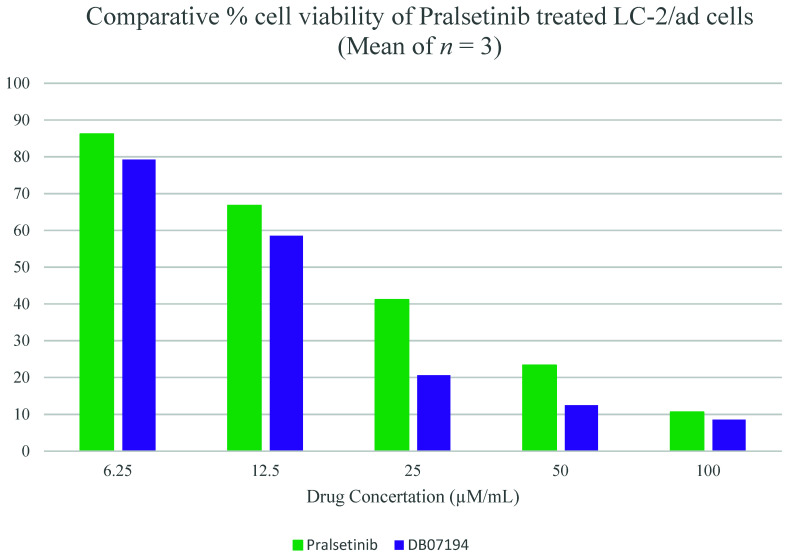
In vitro evaluation of DB07194 inhibitory activity against LC-2/ad cell lines.

Finally, the inhibitory activity of pralsetinib and DB07194 was assessed against LC-2/ad cell lines using a colorimetric MTT assay. The compounds were examined and compared at five different concentrations, 6.25, 12.5, 25, 50, and 100 µM/mL, respectively. The experiment was performed in triplet to overcome the experimental error. Figure 6 and supplementary Table S7 (see Supplementary Materials) represent the comparative cell viability upon treatment with pralsetinib and DB07194. A similar pattern of inhibition was observed between pralsetinib and DB07194 at concentrations 6.25 and 12.5 µM/mL. Interestingly, a sudden rise in the inhibition of LC-2/ad cell using DB07194 was noted at 25 µM/mL, whereas only a smaller variation was observed on using pralsetinib at the same concentration. The inhibitory action does not show much deviation after 50 µM/mL of drugs, which shows the saturation level of inhibition. Overall, LC-2/ad showed higher sensitivity to DB07194 (IC_50_ = 12.48 µM) than pralsetinib (IC_50_ = 23.31 µM). Consistent with its anti-cancer activity, both pralsetinib and DB07194 can decrease the cell viability more significantly than control. Moreover, the anticancer property of DB07194 reveals different pharmacological properties of the compound tested earlier in the experiments as an SYK inhibitor [48,49]. Subsequently, one-way ANOVA analysis was implemented to examine the significance of the difference in cell viability between the control and drug-treated samples. A *p*-value of less than 0.001 is observed between the control and drug-treated sample. This highlights the statistical significance of the experimental data carried out in our study. In addition, no literature evidence has been reported on the toxicity and side effects of the compound. Hence, the toxicity of the hit molecule was also assessed using the ProTox II server and compared against pralsetinib [50]. For instance, predicted LD50 values of pralsetinib and DB07194 were found to be 800 mg/kg and 681 mg/kg, respectively, and thus fall into the class four (slightly toxic) category of compounds. All these data are evidence that the identified hit molecule, DB07194, belongs to an experimental subset of the DrugBank database, displaying favorable drug-like properties and potential progression into clinical application. Thus, it could be considered for the treatment of RET-positive NSCLC, a contrast to the properties for which it was originally designed and synthesized.

## 4. Limitations and Future Prospective

Acquired drug resistance is the major restraint among RET inhibitors resulting in reduced efficacy of drugs in NSCLC patients. Therefore, we examined the activity of the hit compound against 11 different RET mutations in this study. Although the identified hit can demonstrate potent activity against solvent front mutations (G810C, G810V, and M918T), experimental validation of the compound activity using mutant cell lines is certainly needed to validate this finding. The toxicity studies of this compound either by in vivo micronucleus assays or in vitro genotoxicity assays are also interesting future directions. The in vitro activity of the compound identified by the LC-2/ad cell line in our study opens up a new avenue for biologists to explore the synergistic activity of the compound. Finally, the results of our study will facilitate hit-to-lead optimization to reach novel compounds with economic value in the near future.

## 5. Conclusions

The current research focuses on the identification of potential candidates against RET and its associated solvent frontline mutations using high-throughput drug discovery strategies. Different pharmacophore models were employed along with docking, Tanimoto coefficient calculations, rescoring with RF score, and MM-GBSA to deduce the structural characteristics and binding poses that govern the activity of inhibitors against the RET receptor. Comparative DFT analysis was carried out, and it was observed that the lead molecules exhibited a lower energy gap than pralsetinib, depicting more inhibitory potential against the protein. Furthermore, the stability and flexibility of the complex system were analyzed using molecular dynamics for 200 ns. The interaction surface of the protein Val871–Asp898 was found to be conserved, and contained a series of important residues and thus formed hydrogen bonds with the lead molecules. Moreover, the aminopyrimidine group in DB07194 facilitated inhibition of both native and mutant forms of RET with higher binding free energy than pralsetinib. Ultimately, the cell line studies proved the efficiency of the predicted RET inhibitor, showing a lower required minimal drug concentration for inhibiting the RET protein than the existing FDA-approved drug pralsetinib. J.L. Kutok’s patent, namely WO2017223422A1 also mentions the chemical compound DB07194 as a potential third chemotherapeutic agent used in combinations with phosphoinositide 3-kinase inhibitors for cancer treatment. Taken together, the results from our study provide a new gateway for developing DB07194 as a potent anticancer agent targeting RET protein and overcoming the RET-associated solvent front mutations.

## Figures and Tables

**Table 1:** Docking and rescoring evaluation of lead molecules using different strategies.

S. No	DrugBank ID	XP GScore (kcal/mol)	RF Score	Tanimoto Coefficient (T_c_)
Reference	Pralsetinib	−7.79	5.962	1.000
1	DB07194	−9.556	5.974	0.418
2	DB08583	−9.012	6.235	0.423
3	DB12672	−9.579	6.986	0.435
4	DB03496	−10.791	7.108	0.48
5	DB07606	−9.291	7.099	0.502
6	DB12848	−8.066	5.978	0.405
7	DB11982	−9.001	6.644	0.436
8	DB04751	−8.395	6.955	0.432
9	DB07981	−8.117	6.054	0.484
10	DB07248	−8.133	6.268	0.413
11	DB08052	−9.398	7.098	0.451
12	DB07474	−8.133	6.219	0.447
13	DB11665	−9.034	5.99	0.48
14	DB07382	−9.381	6.071	0.4
15	DB02933	−9.169	6.084	0.429
16	DB04338	−9.691	7.005	0.401
17	DB06852	−9.327	6.589	0.432
18	DB02282	−9.108	6.048	0.436

**Table 2:** The predicted binding free energy of RET-complex structures calculated using MM-GBSA approach.

S. No	DrugBank ID	dG Bind (kcal/mol)	Van der Waal’s Energy (kcal/mol)	Ligand Strain Energy (kcal/mol)	Electrostatic Potential (kcal/mol)	Lipophilicity (kcal/mol)	Covalent Interaction (kcal/mol)	Solvation Energy
Reference	Pralsetinib	−63.348	−58.387	6.20432	−12.472	−19.969	−0.4283	37.3355
1	DB07194	−69.235	−46.133	3.22011	−46.443	−17.32	2.77209	40.7179
2	DB08583	−61.769	−48.94	6.4073	−11.888	−18.303	7.34065	36.8761
3	DB12672	−60.017	−51.402	5.56562	−23.095	−20.949	3.90398	33.2395
4	DB03496	−55.502	−46.937	4.2076	−21.81	−20	2.56844	31.9131
5	DB07606	−55.367	−42.62	8.36976	−10.801	−25.237	4.99298	28.2519
6	DB12848	−55.33	−43.57	5.76015	−27.463	−23.935	0.53004	21.6655
7	DB11982	−55.102	−41.865	5.45963	−30.654	−16.496	2.69688	30.5778
8	DB04751	−55.091	−53.348	13.4545	−16.888	−18.303	11.9706	24.1207

**Table 3:** Total binding energy of the lead molecules against RET protein obtained from MM-PBSA analysis.

S. No	DrugBank ID	Binding Energy (kJ/mol)	Van der Waal Energy (kJ/mol)	Electrostatic Energy (kJ/mol)	Polar Solvation Energy (kJ/mol)	SASA Energy (kJ/mol)
Reference	Pralsetinib	−9.445 ± 65.091	−23.022 ± 53.334	−0.074 ± 3.936	15.905 ± 55.514	−2.254 ± 6.035
1	DB07194	−111.920 ± 17.179	−141.170 ± 11.926	−13.371 ± 9.680	55.122 ± 16.524	−12.500 ± 1.161
2	DB03496	−74.514 ± 77.458	−73.039 ± 94.546	1.261 ± 3.289	2.851 ± 61.775	−5.587 ± 7.630
3	DB11982	−37.949 ± 42.565	−90.713 ± 51.388	−43.922 ± 25.645	106.888 ± 52.148	−10.202 ± 6.052

## Data Availability

Not applicable.

## Supplementary Materials

The following contents are available at https://www.mdpi.com/article/10.3390/pharmaceutics13111775/s1. Table S1. List of existing RET inhibitors retrieved from literature. Table S2. Rescoring and structure similarity analysis of screened compounds obtained using pharmacophore based strategy. Table S3. Rescoring and structure similarity analysis of screened compounds obtained using e-pharmacophore based strategy. Table S4. Rescoring and structure similarity analysis of screened compounds obtained using receptor cavity based strategy. Table S5. Interaction and pharmacokinetic evaluation of screened RET inhibitors. Table S6. Mutational analysis of predicted compounds using docking and MM-GBSA studies. Table S7. Examination of cell viability at different drug concentrations (µM/mL) against LC-2/ad cell line. Figure S1. Binding mode analysis of reference compound (a) Pralsetinib, (b) DB07194, (C) DB03496 and (d) DB11982 with the target RET protein. Figure S2. Comparative interaction analysis of (a) crizotinib and (b) Sorafenib with RET protein.
