# Supplementary material for: Discovery of a Potent Candidate for RET-Specific Non-Small-Cell Lung Cancer—A Combined In Silico and In Vitro Strategy
Source: Pharmaceutics. 2021 Oct 24;13(11):1775. doi: 10.3390/pharmaceutics13111775 (PMC8619101; doi:10.3390/pharmaceutics13111775)
Supplement: Supplementary file 1 [file pharmaceutics-13-01775-s001.zip › pharmaceutics-1379056-supplementary.pdf]

pharmaceutics

MDPI

Supplementary Materials: Discovery of a Potent Candidate for RET-Specific Non-Small-Cell Lung Cancer—A Combined in Silico and In Vitro Strategy

Priyanka Ramesh, Woong-Hee Shin and Shanthi Veerappapillai

Table S1. List of existing RET inhibitors retrieved from literature.

S. No	Compounds Name	PubChem ID	Active / Inactive	IC_{50} (μM)
1	Pralsetinib	129073603	Active	0.4
2	Lenvatinib	9823820	Active	1.5
3	RXDX-105	56846693	Active	3
4	Vandetanib	3081361	Active	4
5	Crizotinib	11626560	Active	5
6	Sunitinib	5329102	Active	6.6
7	Sorafenib	216239	Inactive	7.3
8	Regorafenib	11167602	Inactive	8.8
9	AD80	71578106	Inactive	9
10	Cabozantinib	25102847	Inactive	11
11	Apatinib	11315474	Inactive	13
12	Ponatinib	24826799	Inactive	25.8

Table S2. Rescoring and structure similarity analysis of screened compounds obtained using pharmacophore based strategy.

S. No	Compounds ID	RF Score	Tanimoto Coefficient (T_C)	S. No	Compounds ID	RF Score	Tanimoto Coefficient (T_C)
Reference	Pralsetinib	5.962	1	13	DB01645	7.098	0.285
1	DB00179	6.097	0.139	14	DB01852	7.085	0.337
2	DB00242	7.098	0.431	15	DB02103	7.098	0.417
3	DB00317	5.959	0.385	16	DB02224	7.06	0.375
4	DB00905	6.032	0.239	17	DB02282	6.048	0.436
5	DB01094	7.065	0.327	18	DB02359	6.165	0.449
6	DB01102	6.113	0.189	19	DB02375	7.08	0.349
7	DB01148	7.108	0.414	20	DB02537	6.179	0.306
8	DB01193	6.246	0.294	21	DB02550	6.005	0.487
9	DB01250	6.19	0.173	22	DB02754	6.219	0.445
10	DB01438	5.977	0.157	23	DB02915	6.588	0.336
11	DB01558	6.048	0.363	24	DB02933	6.084	0.429
12	DB01594	6.006	0.413	25	DB02947	6.207	0.428
26	DB02984	5.958	0.334	42	DB03980	6.664	0.376
27	DB03037	6.044	0.307	43	DB04054	5.984	0.472
28	DB03097	5.976	0.347	44	DB04101	6.01	0.293
29	DB03267	6.253	0.462	45	DB04196	5.964	0.343
30	DB03312	5.959	0.385	46	DB04216	7.102	0.348
31	DB03365	6.049	0.322	47	DB04244	5.945	0.378
32	DB03467	7.065	0.295	48	DB04254	5.994	0.475
33	DB03496	7.108	0.48	49	DB04274	7.09	0.285
34	DB03716	6.075	0.424	50	DB04338	7.005	0.401
35	DB03804	5.952	0.426	51	DB04357	5.966	0.343
36	DB03809	6.164	0.43	52	DB04407	5.988	0.339
37	DB03878	6.043	0.363	53	DB04437	5.974	0.273
38	DB03881	7.084	0.475	54	DB04442	6.198	0.293
39	DB03899	6.273	0.422	55	DB04463	7.098	0.402
40	DB03916	6.947	0.345	56	DB04604	6.049	0.45

41	DB03921	6.571	0.318	57	DB04751	6.955	0.432
58	DB04764	6.48	0.332	74	DB07065	6.036	0.464
59	DB04806	6.025	0.401	75	DB07066	7.098	0.433
60	DB04809	6.506	0.226	76	DB07101	6.366	0.322
61	DB04816	5.953	0.226	77	DB07107	6.629	0.327
62	DB06061	6.571	0.432	78	DB07119	5.992	0.141
63	DB06190	7.098	0.262	79	DB07126	7.01	0.368
64	DB06485	6.793	0.32	80	DB07146	5.987	0.434
65	DB06609	7.098	0.343	81	DB07163	5.95	0.433
66	DB06649	6.859	0.183	82	DB07170	6.588	0.401
67	DB06814	6.354	0.231	83	DB07173	6.149	0.445
68	DB06837	5.957	0.412	84	DB07175	6.326	0.332
69	DB06852	6.589	0.432	85	DB07194	5.974	0.418
70	DB06999	6.003	0.424	86	DB07202	6.706	0.425
71	DB07000	6.124	0.435	87	DB07248	6.268	0.413
72	DB07024	7.082	0.485	88	DB07250	6.507	0.357
73	DB07054	6.665	0.331	89	DB07252	6.584	0.379
90	DB07266	6.004	0.516	106	DB07602	6.046	0.339
91	DB07268	6.253	0.292	107	DB07606	7.099	0.502
92	DB07303	6.001	0.272	108	DB07618	6.045	0.51
93	DB07335	7.098	0.401	109	DB07648	6.105	0.402
94	DB07352	7.085	0.29	110	DB07653	5.952	0.452
95	DB07382	6.071	0.4	111	DB07655	6.148	0.389
96	DB07410	6.086	0.326	112	DB07662	6.117	0.314
97	DB07474	6.038	0.447	113	DB07686	7.096	0.379
98	DB07481	6.932	0.343	114	DB07698	6.097	0.371
99	DB07488	5.999	0.349	115	DB07715	5.959	0.261
100	DB07489	6.282	0.353	116	DB07750	6.286	0.304
101	DB07495	6.577	0.435	117	DB07751	6.018	0.286
102	DB07504	6.014	0.294	118	DB07761	6.536	0.286
103	DB07525	6.98	0.237	119	DB07766	5.961	0.459
104	DB07545	6.166	0.32	120	DB07791	6.319	0.403
105	DB07546	6.1	0.447	121	DB07795	7.095	0.325
122	DB07809	6.213	0.262	138	DB08150	6.223	0.357
123	DB07832	6.095	0.346	139	DB08178	7.025	0.345
124	DB07877	6.024	0.462	140	DB08182	6.974	0.362
125	DB07926	6.01	0.31	141	DB08208	5.97	0.302
126	DB07942	7.093	0.24	142	DB08218	6.342	0.342
127	DB07981	6.054	0.484	143	DB08230	7.095	0.309
128	DB07984	6.149	0.484	144	DB08241	6.935	0.37
129	DB08004	6.017	0.393	145	DB08267	6.569	0.372
130	DB08026	6.584	0.351	146	DB08313	6.161	0.309
131	DB08052	7.098	0.451	147	DB08314	7.098	0.229
132	DB08053	7.098	0.443	148	DB08325	6.955	0.397
133	DB08067	6.348	0.365	149	DB08350	6.156	0.382
134	DB08073	5.991	0.393	150	DB08392	5.967	0.452
135	DB08112	6.143	0.321	151	DB08393	5.952	0.445
136	DB08130	6.048	0.377	152	DB08395	5.992	0.477
137	DB08136	6.937	0.282	153	DB08436	6.272	0.453
154	DB08442	6.226	0.345	170	DB08860	6.597	0.447
155	DB08462	6.273	0.3	171	DB08893	6.161	0.26
156	DB08466	6.579	0.1	172	DB08907	6.804	0.402
157	DB08470	6.975	0.153	173	DB08916	5.959	0.434
158	DB08517	7.065	0.3	174	DB08974	6.146	0.26
159	DB08527	5.964	0.412	175	DB09330	6.549	0.448
160	DB08539	6.108	0.45	176	DB11259	7.09	0.321
161	DB08557	6.882	0.493	177	DB11336	7.008	0.262
162	DB08564	6.208	0.331	178	DB11656	6.992	0.366
163	DB08568	5.993	0.335	179	DB11665	5.98	0.48
164	DB08569	5.982	0.341	180	DB11865	7.069	0.488

165	DB08572	6.061	0.357	181	DB11919	7.01	0.256
166	DB08583	6.235	0.423	182	DB11958	6.997	0.475
167	DB08673	6.18	0.234	183	DB11967	7.098	0.422
168	DB08694	6.156	0.458	184	DB11982	6.644	0.436
169	DB08787	7.098	0.301	185	DB12039	6.532	0.315
186	DB12174	5.977	0.372	198	DB12937	7.087	0.383
187	DB12183	6.311	0.417	199	DB12941	7.098	0.384
188	DB12218	6.005	0.43	200	DB12966	5.956	0.363
189	DB12387	6.997	0.543	201	DB12974	6.128	0.376
190	DB12433	6.084	0.375	202	DB13059	6.077	0.479
191	DB12566	6.961	0.387	203	DB13074	6.078	0.379
192	DB12611	6.312	0.513	204	DB13174	5.991	0.275
193	DB12672	6.986	0.435	205	DB13203	6.066	0.448
194	DB12687	6.536	0.407	206	DB13837	5.964	0.4
195	DB12746	6.496	0.298	207	DB13983	7.085	0.384
196	DB12848	5.978	0.405	208	DB14008	7.085	0.332
197	DB12927	6.414	0.409				

Table S3. Rescoring and structure similarity analysis of screened compounds obtained using e-pharmacophore based strategy.

S. No	Compounds ID	RF Score	Tanimoto Coefficient (Tc)	S. No	Compounds ID	RF Score	Tanimoto Coefficient (Tc)
Reference	Pralsetinib	5.962	1	13	DB06190	7.098	0.345
1	DB00179	6.097	0.139	14	DB06852	6.589	0.376
2	DB01102	6.113	0.189	15	DB07101	6.366	0.302
3	DB02282	6.048	0.238	16	DB07107	6.629	0.401
4	DB02550	6.005	0.344	17	DB07126	7.01	0.339
5	DB02933	6.084	0.436	18	DB07175	6.326	0.376
6	DB03365	6.049	0.487	19	DB07194	5.974	0.432
7	DB03496	7.108	0.387	20	DB07248	6.268	0.363
8	DB03916	6.947	0.429	21	DB07252	6.584	0.262
9	DB03980	6.664	0.396	22	DB07268	6.253	0.445
10	DB04338	7.005	0.472	23	DB07303	6.001	0.432
11	DB04407	5.988	0.322	24	DB07382	6.071	0.337
12	DB04751	6.955	0.48	25	DB07474	6.038	0.393
26	DB07489	6.282	0.322	42	DB08350	6.156	0.447
27	DB07504	6.014	0.327	43	DB08568	5.993	0.353
28	DB07545	6.166	0.368	44	DB08569	5.982	0.294
29	DB07606	7.099	0.462	45	DB08583	6.235	0.274
30	DB07686	7.096	0.332	46	DB08860	6.597	0.32
31	DB07698	6.097	0.418	47	DB09330	6.549	0.502
32	DB07791	6.319	0.394	48	DB11665	5.98	0.426
33	DB07981	6.054	0.246	49	DB11958	6.997	0.379
34	DB08004	6.017	0.338	50	DB11967	7.098	0.371
35	DB08026	6.584	0.413	51	DB11982	6.644	0.304
36	DB08052	7.098	0.379	52	DB12218	6.005	0.403
37	DB08182	6.974	0.292	53	DB12387	6.997	0.387
38	DB08208	5.97	0.272	54	DB12672	6.986	0.389
39	DB08241	6.935	0.404	55	DB12848	5.978	0.401
40	DB08267	6.569	0.441	56	DB12941	7.098	0.484
41	DB08325	6.955	0.4	57	DB01182	6.174	0.39
58	DB02197	6.15	0.258	74	DB08532	7.098	0.36
59	DB03072	6.103	0.393	75	DB08613	6.744	0.382
60	DB04588	7.085	0.351	76	DB09199	6.48	0.337
61	DB05575	6.027	0.451	77	DB09292	5.952	0.309
62	DB06589	5.972	0.416	78	DB11832	6.865	0.353
63	DB06967	6.065	0.362	79	DB12432	5.969	0.332
64	DB07245	6.203	0.302	80	DB12535	6.358	0.335
65	DB07247	6.018	0.37	81	DB02830	6.5	0.341

66	DB07649	6.867	0.375	82	DB03137	5.994	0.423
67	DB07755	6.963	0.372	83	DB03984	6.029	0.456
68	DB07853	6.568	0.368	84	DB06925	6.079	0.447
69	DB07889	6.302	0.397	85	DB07149	6.218	0.291
70	DB07993	6.508	0.526	86	DB07235	7.098	0.259
71	DB08248	7.01	0.382	87	DB07333	6.028	0.448
72	DB08356	5.951	0.309	88	DB07379	6.962	0.359
73	DB08459	6.187	0.375	89	DB07512	7.098	0.48
90	DB07812	6.08	0.324	97	DB08513	6.035	0.543
91	DB07991	6.021	0.453	98	DB08514	6.229	0.351
92	DB08054	5.992	0.419	99	DB08537	6.149	0.434
93	DB08300	7.062	0.475	100	DB11645	6.126	0.435
94	DB08349	6.144	0.422	101	DB11764	6.951	0.405
95	DB08457	6.072	0.436	102	DB11798	6.114	0.384
96	DB08460	6.165	0.43	103	DB13083	6.065	0.385

Table S4. Rescoring and structure similarity analysis of screened compounds obtained using receptor cavity based strategy.

S. No	Compounds ID	RF Score	Tanimoto Coefficient (T_C)	S. No	Compounds ID	RF Score	Tanimoto Coefficient (T_C)
Reference	Pralsetinib	5.962	1	14	DB01645	7.098	0.285
1	DB00179	6.097	0.139	15	DB01852	7.085	0.337
2	DB00242	7.098	0.431	16	DB02103	7.098	0.417
3	DB00299	7.098	0.314	17	DB02197	6.15	0.344
4	DB00495	5.979	0.382	18	DB02224	7.06	0.375
5	DB00800	6.014	0.305	19	DB02282	6.048	0.436
6	DB00841	5.983	0.161	20	DB02359	6.165	0.449
7	DB00842	5.97	0.314	21	DB02375	7.08	0.349
8	DB00905	6.032	0.239	22	DB02500	5.954	0.245
9	DB01094	7.065	0.327	23	DB02568	5.97	0.315
10	DB01182	6.174	0.238	24	DB02733	6.965	0.461
11	DB01250	6.19	0.173	25	DB02754	6.219	0.445
12	DB01438	5.977	0.157	26	DB02830	6.5	0.387
13	DB01558	6.048	0.363	27	DB02898	7.01	0.313
28	DB02915	6.588	0.336	44	DB03735	6.496	0.387
29	DB02933	6.084	0.429	45	DB03763	6.034	0.324
30	DB02947	6.207	0.428	46	DB03804	5.952	0.426
31	DB02984	5.958	0.334	47	DB03809	6.164	0.43
32	DB02985	6.039	0.226	48	DB03878	6.043	0.363
33	DB03074	7.01	0.273	49	DB03881	7.084	0.475
34	DB03172	6.17	0.412	50	DB03899	6.273	0.422
35	DB03216	6.877	0.383	51	DB03916	6.947	0.345
36	DB03267	6.253	0.462	52	DB03921	6.571	0.318
37	DB03285	6.059	0.177	53	DB03980	6.664	0.376
38	DB03312	5.959	0.385	54	DB04004	6.161	0.334
39	DB03365	6.049	0.322	55	DB04030	6.515	0.468
40	DB03467	7.065	0.295	56	DB04054	5.984	0.472
41	DB03496	7.108	0.48	57	DB04101	6.01	0.293
42	DB03693	6.252	0.265	58	DB04196	5.964	0.343
43	DB03716	6.075	0.424	59	DB04216	7.102	0.348
60	DB04239	6.18	0.338	76	DB05575	6.027	0.363
61	DB04254	5.994	0.475	77	DB06152	6.594	0.19
62	DB04274	7.09	0.285	78	DB06198	5.961	0.362
63	DB04338	7.005	0.401	79	DB06397	6.074	0.204
64	DB04357	5.966	0.343	80	DB06485	6.793	0.32
65	DB04407	5.988	0.339	81	DB06589	5.972	0.445
66	DB04442	6.198	0.293	82	DB06609	7.098	0.343
67	DB04463	7.098	0.402	83	DB06619	6.783	0.398
68	DB04546	7.098	0.398	84	DB06649	6.859	0.183

69	DB04583	5.996	0.198	85	DB06732	6.855	0.252
70	DB04662	6.955	0.417	86	DB06814	6.354	0.231
71	DB04707	7.066	0.301	87	DB06837	5.957	0.412
72	DB04751	6.955	0.432	88	DB06852	6.589	0.432
73	DB04764	6.48	0.332	89	DB06915	7.085	0.094
74	DB04809	6.506	0.226	90	DB06977	6.02	0.367
75	DB04961	6.448	0.299	91	DB07000	6.124	0.435
92	DB07008	7.098	0.298	108	DB07335	7.098	0.401
93	DB07009	6.878	0.327	109	DB07352	7.085	0.29
94	DB07119	5.992	0.141	110	DB07362	6.894	0.424
95	DB07132	6.231	0.427	111	DB07364	6.025	0.379
96	DB07158	6.233	0.347	112	DB07379	6.962	0.441
97	DB07163	5.95	0.433	113	DB07382	6.071	0.4
98	DB07173	6.149	0.445	114	DB07426	6.243	0.237
99	DB07175	6.326	0.332	115	DB07435	5.958	0.255
100	DB07194	5.974	0.418	116	DB07444	6.538	0.381
101	DB07202	6.706	0.425	117	DB07474	6.038	0.447
102	DB07248	6.268	0.413	118	DB07524	7.098	0.206
103	DB07253	6.425	0.383	119	DB07525	6.98	0.237
104	DB07268	6.253	0.292	120	DB07545	6.166	0.32
105	DB07284	6.881	0.231	121	DB07606	7.099	0.502
106	DB07303	6.001	0.272	122	DB07618	6.045	0.51
107	DB07333	6.028	0.404	123	DB07648	6.105	0.402
124	DB07686	7.096	0.379	140	DB08026	6.584	0.351
125	DB07698	6.097	0.371	141	DB08049	7.098	0.3
126	DB07715	5.959	0.261	142	DB08052	7.098	0.451
127	DB07766	5.961	0.459	143	DB08054	5.992	0.416
128	DB07789	6.082	0.451	144	DB08070	6.242	0.178
129	DB07795	7.095	0.325	145	DB08073	5.991	0.393
130	DB07812	6.08	0.387	146	DB08114	6.598	0.149
131	DB07829	7.086	0.213	147	DB08130	6.048	0.377
132	DB07854	7.012	0.27	148	DB08136	6.937	0.282
133	DB07926	6.01	0.31	149	DB08178	7.025	0.345
134	DB07942	7.093	0.24	150	DB08182	6.974	0.362
135	DB07981	6.054	0.484	151	DB08191	6.473	0.265
136	DB07991	6.021	0.39	152	DB08208	5.97	0.302
137	DB07993	6.508	0.258	153	DB08218	6.342	0.342
138	DB07997	7.085	0.215	154	DB08230	7.095	0.309
139	DB08004	6.017	0.393	155	DB08248	7.01	0.375
156	DB08251	6.005	0.33	172	DB08532	7.098	0.353
157	DB08349	6.144	0.526	173	DB08557	6.882	0.493
158	DB08350	6.156	0.382	174	DB08562	6.513	0.162
159	DB08353	6.052	0.408	175	DB08568	5.993	0.335
160	DB08378	6.515	0.202	176	DB08583	6.235	0.423
161	DB08395	5.992	0.477	177	DB08660	5.947	0.258
162	DB08432	5.96	0.376	178	DB08661	5.958	0.391
163	DB08436	6.272	0.453	179	DB08673	6.18	0.234
164	DB08442	6.226	0.345	180	DB08694	6.156	0.458
165	DB08462	6.273	0.3	181	DB08893	6.161	0.26
166	DB08466	6.579	0.1	182	DB11259	7.09	0.321
167	DB08470	6.975	0.153	183	DB11336	7.008	0.262
168	DB08513	6.035	0.337	184	DB11337	7.008	0.233
169	DB08514	6.229	0.309	185	DB11645	6.126	0.359
170	DB08517	7.065	0.3	186	DB11656	6.992	0.366
171	DB08527	5.964	0.412	187	DB11665	5.98	0.48
188	DB11909	6.433	0.501	204	DB12855	6.386	0.309
189	DB11982	6.644	0.436	205	DB12894	7.147	0.369
190	DB12028	7.095	0.355	206	DB12919	6.044	0.367
191	DB12039	6.532	0.315	207	DB12927	6.414	0.409
192	DB12156	6.469	0.404	208	DB12937	7.087	0.383

193	DB12353	7.098	0.393	209	DB12957	7.07	0.361
194	DB12383	6.018	0.376	210	DB13203	6.066	0.448
195	DB12432	5.969	0.351	211	DB13757	6.152	0.234
196	DB12433	6.084	0.375	212	DB13837	5.964	0.4
197	DB12438	7.098	0.354	213	DB13983	7.085	0.384
198	DB12450	6.089	0.158	214	DB14008	7.085	0.332
199	DB12531	7.087	0.373	215	DB14028	5.965	0.281
200	DB12566	6.961	0.387	216	DB14086	6.546	0.315
201	DB12672	6.986	0.435	217	DB14109	6.888	0.247
202	DB12746	6.496	0.298	218	DB14122	6.047	0.206
203	DB12848	5.978	0.405	219	DB14127	6.212	0.249

Table S5. Interaction and pharmacokinetic evaluation of screened RET inhibitors.

S. No	DrugBank ID	Interaction analysis		Pharmacokinetic parameters				
		**Type of Interaction**	**Interaction pattern**	**Stars**	**CNS**	**HOA**	**QPlogS**	**QlogBB**
1	Pralsetinib	Hydrogen Bond	NH … ALA807<br>ALA807 … N<br>SER811 … N	0	−2	2	−6.287	−0.469
2	DB07194	Hydrogen Bond	NH … ASP892<br>OH … ASN878	0	−2	3	−6.189	−1.251
3	DB03496	Hydrogen Bond	OH … ARG878<br>ALA807 … O	0	1	3	−3.972	−0.521
4	DB11982	Salt Bridge<br>Hydrogen Bond	ASP892 … $N^{+}H$<br>OH … ARG878	0	−2	3	−4.901	−1.18

Table S6. Mutational analysis of predicted compounds using docking and MM-GBSA studies.

Glide XP Gscore (kcal/mol)												
**S. No**	**DrugBank ID**	**G810C**	**G810R**	**G810S**	**G810V**	**M918T**	**V738A**	**V804E**	**V804L**	**V804M**	**Y806C**	**Y806N**
Reference	Pralsetinib	−3.69	−3.356	−6.69	−5.684	−4.85	−4.555	−6.682	−8.067	−7.625	−5.764	−4.81
1	DB07194	−6.548	−6.267	−5.918	−6.895	−8.196	−7.725	−7.493	−5.773	−8.503	−7.917	−9.133
2	DB03496	ND	−10.18	ND	ND	−7.818	−5.55	ND	−5.967	−4.001	−7.955	−7.553
3	DB11982	−6.363	−6.773	−8.536	−4.374	−7.56	−4.877	−5.138	−5.44	−9.157	−7.945	−8.494
**dG Bind (kcal/mol)**												
**S. No**	**DrugBank ID**	**G810C**	**G810R**	**G810S**	**G810V**	**M918T**	**V738A**	**V804E**	**V804L**	**V804M**	**Y806C**	**Y806N**
Reference	Pralsetinib	−73	−77.15	−83.23	−61.59	−50.29	−67.41	−92.35	−83.72	−94.27	−77.27	−68.39
1	DB07194	−79.58	−63.42	−65.68	−80.54	−74.11	−46.27	−77.21	−78.33	−60.63	−76.22	−63.32
2	DB03496	ND	−80.79	ND	ND	−87.16	−66.54	ND	−61.39	−54.001	−61.69	−58.19
3	DB11982	−67.74	−69.5	−68.34	−64.01	−47.06	−49.53	−69.96	−70.25	−66.99	−65.77	−67.71

ND: Compound got eliminated during docking analysis.

Table S7. Examination of cell viability at different drug concentrations (μM/ml) against LC-2/ad cell line.

	Pralsetinib						DB07194					
**S. No**	**Control**	**6.25**	**12.5**	**25**	**50**	**100**	**Control**	**6.25**	**12.5**	**25**	**50**	**100**
1	100	86.18	66.74	41.14	23.31	10.61	100	79.22	66.74	20.59	12.48	8.55
2	100	84.5	69.53	39.59	24.95	9.6	100	83.5	68.5	18.9	12.23	8.667
3	100	85.8	67.55	42.69	22.1	11.3	100	81.96	64.9	21.3	13.05	9.63
Sum	300	256.48	203.82	123.42	70.36	31.51	300	244.68	200.14	60.79	37.76	26.853
Average	100	85.49	67.94	41.14	23.45	10.50	100	81.56	66.71	20.26	12.58	8.95
Variance	0	0.77	2.05	2.40	2.04	0.73	0	4.69	3.24	1.52	0.17	0.34
P-value			5.47×10^{-18}						5.95×10^{-18}			

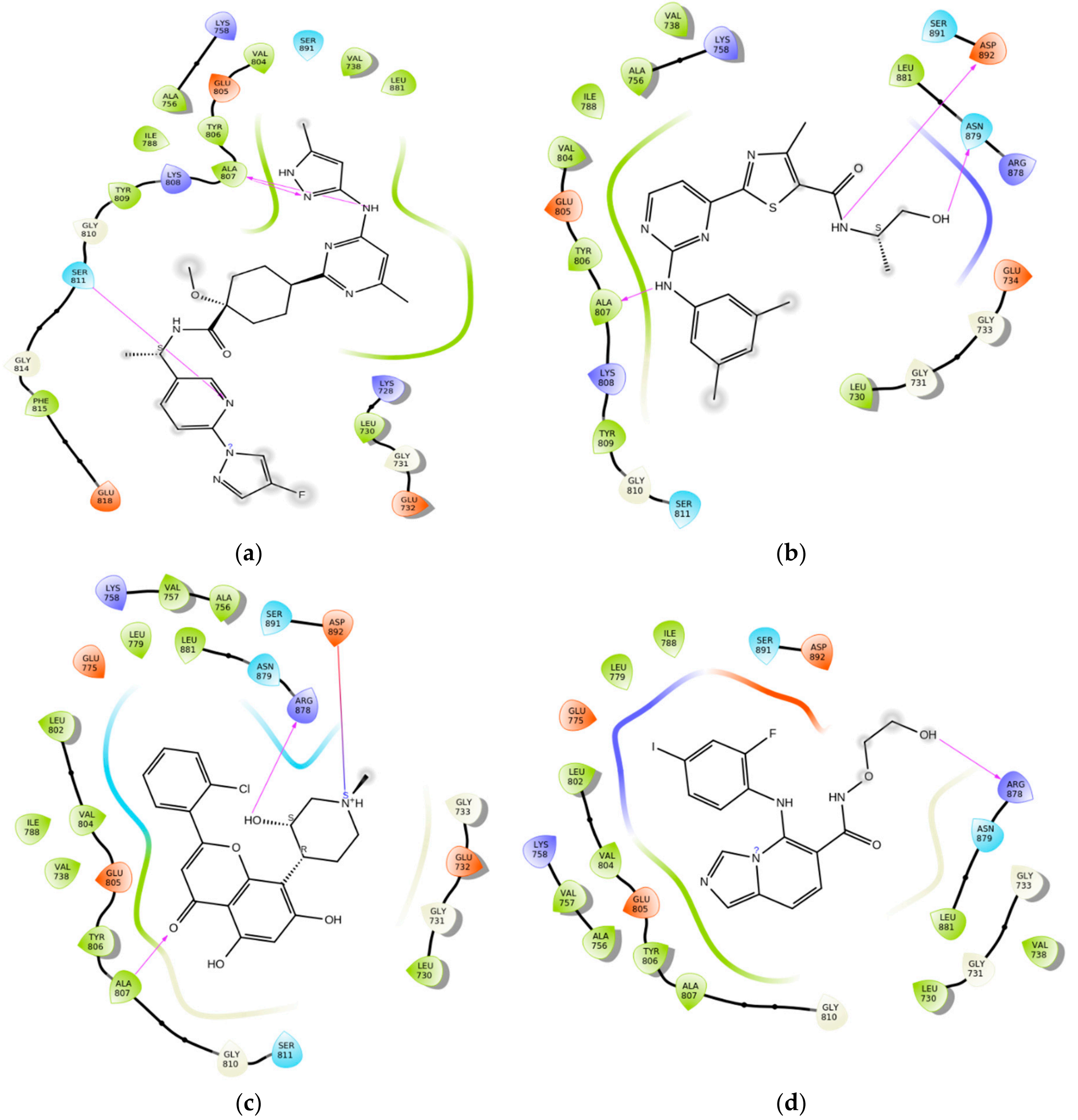

Figure S1. Binding mode analysis of reference compound (**a**) Pralsetinib, (**b**) DB07194, (**c**) DB03496 and (**d**) DB11982 with the target RET protein.

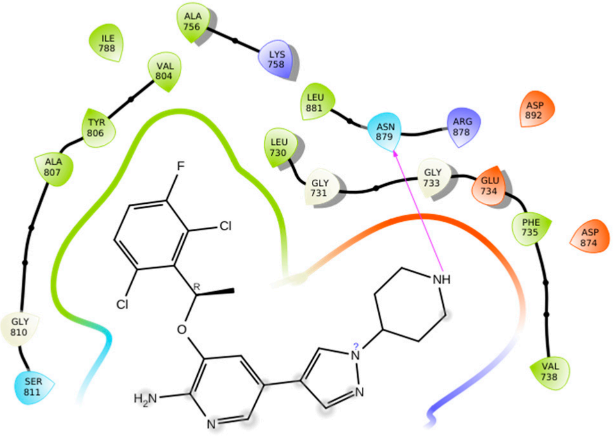

(**a**)

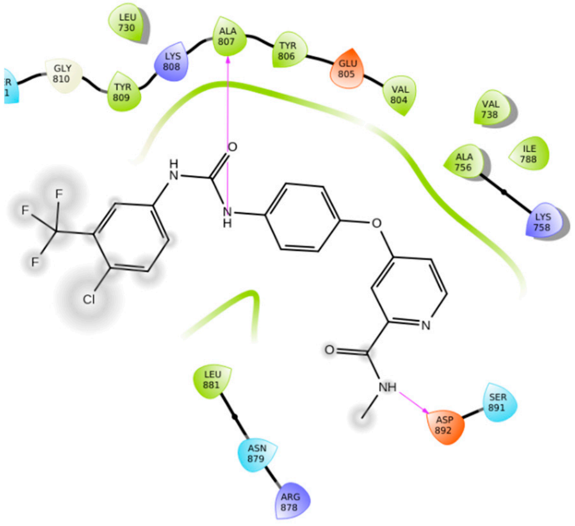

(**b**)

Figure S2. Comparative interaction analysis of (**a**) crizotinib and (**b**) Sorafenib with RET protein.
